# Draft genome sequences of four phosphate-solubilizing microbes of the family *Burkholderiaceae* isolated from tailings from the former Pea Ridge Mine in Washington County, MO, USA

**DOI:** 10.1128/mra.01144-25

**Published:** 2025-11-12

**Authors:** Prince Oppong Amoh, Abiodun E. Idowu, Elizabeth R. Grube, Melanie R. Mormile, Hunter W. Schroer

**Affiliations:** 1 Civil, Architectural, and Environmental Engineering Department, Missouri University of Science and Technology 548058, Rolla, Missouri, USA; 2 Biological Sciences Department, Missouri University of Science and Technology 14717, Rolla, Missouri, USA; University of Manitoba, Winnipeg, Manitoba, Canada

**Keywords:** phosphate-solubilizing microbes, organic acids, plant growth promotors, bioleaching

## Abstract

We present draft genome sequences of four Gram-negative phosphate-solubilizing microbes isolated from Pea Ridge Mine tailings in Missouri, USA. Belonging to the family *Burkholderiaceae*, these isolates exhibit a phosphate-mineral-dissolving phenotype in liquid and solid cultures. Genome sizes span 5.6–9.7 Mb, with GC contents of 61.6%–67.0%.

## ANNOUNCEMENT

Phosphate-solubilizing microorganisms (PSMs) are ubiquitous in the environment, including legacy mine sites (1). PSMs contribute to biogeochemical transformations through the secretion of protons or organic acids, facilitating the solubilization of mineral-bound phosphate, including minerals containing rare earth oxides (2–4). Given the growing demand for rare earth elements, there is increasing interest in microbial strategies for sustainable extraction from unconventional sources, such as using PSMs for leaching of rare earth-phosphate minerals (5, 6). The Pea Ridge Mine, located 13.8 km southeast of Sullivan in Washington County, MO (38°7′33″ N, 91°2′50″ W), is a decommissioned iron deposit enriched in such rare earth-phosphate minerals (7). Accordingly, we collected water and soil samples from seven locations within the former Pea Ridge Mine tailings piles in September 2024 to isolate PSMs. We plated 25 µL of 10% wt/vol slurry suspensions or direct aliquots of the tailings ponds and 10-fold dilutions of each, onto Pikovskaya’s agar (6) and incubated the plates at 30°C for 72 h. Colonies showing distinct Ca_3_(PO_4_)_2_ clearance zones were isolated and purified through at least three successive streak platings.

Four distinct colonies were selected for DNA extraction and sequencing. Single colonies picked from an agar plate were grown overnight in 5 mL of Pikovskaya media (without agar) at 30°C and 100 rpm. Genomic DNA was extracted from 250 µL of media using the DNeasy PowerSoil Pro Kit (Qiagen, Hilden, Germany). Whole-genome sequencing was performed by Plasmidsaurus (San Francisco, CA). Genomic DNA was prepared with Oxford (v.14) library prep chemistry, including minimal fragmentation and no size selection, and sequenced using R10.4.1 flow cells on an Oxford Nanopore PromethION 24 instrument, with base calling performed by the built-in Dorado software. Raw reads were filtered to remove the lowest-quality 5% and down-sampled to 250 Mb using Filtlong (v.0.2.1) then assembled with Miniasm (v.0.3) (8), both with default parameters. Raw reads were also assembled using Flye (v.2.9.1) (9), with the setting for high-quality reads, and the Flye assembly was polished with Medaka (v.1.8.0, default parameters) by referencing the Miniasm assembly. CheckM (v.1.2.2, default parameters) (10) was used to evaluate genome completeness and contamination (Table 1). Genome annotations were performed using the NCBI Prokaryotic Genome Annotation Pipeline (v.6.10, default parameters) (11). The 16S rRNA gene was amplified by PCR using universal primers 27F and 1492R (12). A 50 µL PCR was prepared, which included Q5 High-Fidelity Master Mix (New England Biolabs, Ipswich, MA), 10 µM of each primer, and ~100 to 300 ng of template DNA. PCR products were purified using the QIAquick PCR Purification Kit (Qiagen), and the purified amplicons were Sanger sequenced at the University of Missouri Genomics Core using 27F, 341F, and 1492R primers. The sequences were trimmed and aligned to obtain a full-length consensus 16S sequence using Benchling (2025). Species-level identification was conducted via BLASTn against the NCBI 16S rRNA database (Table 1). The isolates all belonged to the family *Burkholderiaceae* (β-proteobacteria).

**TABLE 1:** Assembly and annotation summaries for isolates

Organism (% identity, closest BLASTn accession #)	Genome assembly accession #	Reads (total #)	Coverage (×)	Completeness (%)	Contamination (%)	Contig (#)	N50 (Mbp)	Size (Mbp)	GC (%)	Genes (#)	CDS (#)	rRNA (#)	tRNA (#)
*Burkholderia vietnamiensis* (100%, NR_118872.1)	GCA_052372895.1	133,348	54	99.57	0.09	6	2.2	6.9	67.0	6,159	5,942	18	69
*Paraburkholderia tropica* (99.93%, NR_028965.1)	GCA_052372875.1	75,639	31	100.0	0.56	4	2.6	7.2	66.0	6,551	6,301	18	70
*Paraburkholderia fungorum* (99.93%, NR_178636.1)	GCA_052372855.1	843,903	95	99.5	3.38	12	2.5	9.7	61.5	8,987	8,742	18	63
*Ralstonia picketti* (100%, NR_114126.1)	GCA_052372795.1	137,651	49	99.97	0.17	14	1.2	5.6	63.5	5,310	5,171	9	53

These isolates represent promising candidates for future investigation into the genetic and metabolic basis of phosphate mineral solubilization, with possible applications for bioleaching of apatite-rich, low-grade minerals.

## Data Availability

This genome sequencing project has been deposited at NCBI. The assembled and annotated genomes are deposited at the following links: GCA_052372895.1, GCA_052372875.1, GCA_052372855.1 and GCA_052372795.1. The 16S rRNA sequences are deposited at GenBank at the following links: PX441982, PX441983, PX441984 and PX429572. The raw sequencing reads are deposited in the Sequencing Read Archive at the following links: SRX30648207, SRX30648208, SRX30648209 and SRX30648210.
